# Behavioral Intentions to Donate Blood: The Interplay of Personality, Emotional Arousals, and the Moderating Effect of Altruistic versus Egoistic Messages on Young Adults

**DOI:** 10.3390/bs14080731

**Published:** 2024-08-22

**Authors:** Stefanos Balaskas, Maria Rigou, Michalis Xenos, Andreas Mallas

**Affiliations:** 1 Department of Management Science and Technology, University of Patras, 26334 Patras, Greece; rigou@upatras.gr; 2 Department of Computer Engineering and Informatics, University of Patras, 26504 Patras, Greece; xenos@ceid.upatras.gr (M.X.); mallas@ceid.upatras.gr (A.M.)

**Keywords:** blood donation, message framing, HEXACO, behavioral intention, attitude, emotional arousal, structural equation modeling

## Abstract

Human blood is one of the most valuable and irreplaceable goods in modern medicine. Although its necessity increases daily, one of the most significant challenges we have to overcome is a scarcity of willing blood donors. Volunteer motives and attitudes have been studied for decades, but it is now considered vital to grasp the many aspects that will increase the effectiveness of attracting new blood donors. This study focuses on the impact of emotional arousal produced by advertising messages, as well as the determining role of altruistic and egoistic incentives in deciding behavior. We also incorporated the element of personality to investigate how personality traits influence behavioral intention to donate blood. To this end, a quantitative non-experimental correlational 2 × 2 experimental design (positive vs. negative emotional appeal; altruistic vs. egoistic message) was implemented with the participation of 462 respondents who were shown a total of 12 advertisements (ads) promoting blood donation. The data were analyzed using structural equation modeling, with a focus on the direct impacts on donation intentions, the role of emotional arousals and attitude towards the ads as mediators and the moderating effect of the message. The empirical results of our hypotheses revealed that only Honesty–Humility had a strong direct impact on behavioral intention to donate, while Emotionality and Agreeableness did not have any direct effect. On the other hand, attitudes towards advertisements significantly and directly influenced positive and negative emotional arousals, respectively. Furthermore, if we consider these two variables alone, they can be found to exert a direct impact on BI. Mediation analysis showed that attitudes towards the advertisements and emotional arousals partially mediated the relation between Honesty-Humility and Behavioral Intention, thus confirming partial mediation. With respect to Emotionality and Agreeableness, mediation was found to be full since these factors only affected BI through a mediated path, which confirmed full mediation. Furthermore, the moderation analysis highlighted that the type of message (altruistic vs. egoistic) significantly moderated the relationship between both emotional arousals and BI. In particular, positive emotional arousal’s influence is strengthened when it is aligned with altruistic messages, while negative emotional arousal’s influence is weakened if it follows an altruistic message. These findings illustrate that using positive emotions will be more beneficial for increasing people’s donation intentions than bringing negative ones, which implies that message framing has a hidden impact on donation decisions.

## 1. Introduction

Volunteering has long been one of the driving principles and active forms of community engagement aimed at enhancing people’s quality of life [1,2]. Volunteering and philanthropy are two distinct ideas, yet they both enable us to comprehend the need for well-being among our fellow humans, as well as its impact on happiness and interdependence among members of society. The rise of volunteerism in psychosocial rehabilitation centers reflects the public’s recognition of mental health concerns and the proactive mindset that sets involved citizens apart [3,4]. Volunteering, in general, refers to the voluntary provision of services for a public purpose, and yet, to this day, it constitutes a controversial point as to whether there should be materialistic incentives. Despite this, many are supporters of the idea that paid voluntary work is necessary and legitimate in modern society. Volunteering is a broad notion that can extend from voluntary blood donation to a posthumous donation of human body parts for scientific purposes [4,5,6]. Thus, volunteerism is a fundamental provision via active engagement that capitalizes on the social economy in the current period, as it offers that type of alternative professionalism that provides unity and cohesiveness to badly disconnected social institutions. Individuals willing to offer their services must be educated or receive training appropriate to their specific roles, ensuring they are well prepared for their upcoming duties. The primary and fundamental incentive for social inclusion and engagement is established by supportively obtaining the unanimous inclusion of individuals, regardless of their backgrounds [3,4,6].

It is essential to realize that voluntary blood donation is different from other types of volunteer activities in terms of certain medical and logistical requirements, though it qualifies as volunteering [1,3,7]. Blood donation involves a desire to assist others, in addition to being medically eligible, going through formalities, and caring about health considerations. Unlike generalized forms of volunteering that derive from a wide range of activities to a serious commitment, with blood donation, potential volunteers have to be screened for their health safety, abide by the security protocols, and follow through the process, which has a direct effect on medical outcome. Therefore, based on the above perspective, this form of blood donation serves critical and immediate healthcare needs [8,9,10].

One of the most serious issues that blood services face worldwide is the constant scarcity of blood. Although the need for blood is growing in many countries, the number of blood donations is steadily declining [1,11]. By blood donation, we mean the administration of blood through transfusion and, by extension, the entire operation that deals with the receipt, storage, and disposal of blood and its derivatives [12,13,14]. Voluntary blood donation is a unique gesture of compassion and one of the most significant manifestations of social volunteering and solidarity. It is regarded as the highest expression of kindness for individuals in need of a transfusion since it is a gift of life to the recipient [15,16]. Moreover, promoting voluntary blood donation is essential for a nation to become self-sufficient in blood. Every day, there is a considerable need for blood, attributable to numerous accidents and diseases that afflict humanity [9,13,17]. A dearth or deficit of blood puts the lives of individuals who require blood as a medicinal agent in urgent peril. Knowing what prevents individuals from contributing and what influences them not to donate is critical for establishing and planning donor recruiting methods. Increasing supply requires the recruitment of fresh blood donors to re-transfuse. Recognizing the importance of this issue, national blood donation policy embraces and utilizes non-profit marketing principles, with the ultimate objective of informing and raising awareness among individuals about voluntary blood donation [8,13,18].

Our research aims to investigate the motives that drive people to donate blood. For this purpose, we designed ads that encourage and invite citizens to donate blood. Each ad features a central photo that triggers a specific emotion and a textual message stressing a reason for donating blood. The primary objective of this study is to investigate the impact of emotional arousal from the ads, both positive and negative, on future behavioral intention to donate blood. The aim is to explore the influence of altruistic and egotistic motives, as well as the influence of personality in volunteering behavior, by drawing from contemporary literature and acknowledging the significance of advertising messages in volunteering behavior. A quantitative non-experimental correlational 2 × 2 experiment was conducted, utilizing techniques of analyzing attitudinal and behavioral changes. The 462 participants were shown a total of 12 ads promoting blood donation (3 positive vs. negative emotional appeals, each one in two variations, one with altruistic and one with an egoistic message). Participants belong to the 18–30 age group (young adults) as blood donation campaigns primarily address young audiences that will become donors early in their lives [14,19,20].

The article is structured as follows: Section 2 presents an overview of the relevant research on the factors that influence and motivate individuals to donate blood, as well as the connections and reasoning behind altruistic and egoistic motives in the context of blood donation behavior. We focus our interest on how consumers perceive blood donation advertisements by studying the impact of attitudes towards the ad, as well as the psychological aspect of consumer behavior with the inclusion of personality traits. Section 3 contains a detailed description of the model we developed and tested for our research purposes, followed by a data analysis in Section 4. Section 5 examines and interprets the main findings and indicates relevant limitations. Finally, Section 6 concludes the article and makes recommendations for further research.

## 2. Literature Review

### 2.1. Volunteer Intentions behind Blood Donation Behavior

Currently, volunteerism is regarded as an integral part of civil society that extends beyond the boundaries of capitalism and the government. It seeks equitable cooperation with them to respond more adequately to the complex social demands highlighted by the intricacy and “dangerousness” of today’s social conditions [21,22,23]. The emergence of new demands provides a chance for volunteers to triumph by developing new activities to satisfy social challenges, such as blood donation. Volunteers determine the functions of an active and involved civil society, which enhances social cohesiveness and participatory democracy. Volunteering strengthens the learning of social, communication, and professional skills while also developing new ones [4,6,24].

People are constantly subjected to stimuli that shape their perspectives and attitudes toward various social phenomena such as culture, politics, and philosophy, while moral influences on an individual’s attitudes and behaviors are always changing. Current research on the motives and stimuli of blood donation intentions, on the other hand, suggests that the subject of study does not provide or produce substantial points of discussion and interest as the motives behind future donations are not fully understood and clarified [9,12,18,25]. Furthermore, there is an increasing realization to reevaluate certain aspects of the blood donation process [19,26,27]. Alas, the extant information on the motives and behavioral changes that lead to blood donation is inadequate. Understanding the characteristics of donor groups also provides crucial information for developing effective donor recruiting and retention strategies [28,29]. The investigation and understanding of the attitudes and motives that drive a blood donor is a basic prerequisite for the efficient recruitment of new volunteers, the retention of existing ones, and the conversion of seasonal ones into regular ones [28,30,31]. Studies have demonstrated that when potential blood donors recognize and perceive the increased need for blood, they are much more motivated and willing to donate [26,32]. In addition, the very experience of donating blood can influence the intention to donate since a positive experience significantly affects future behavioral intentions [8,33].

Ignorance of the magnitude of the actual need brought by the lack of blood leads to the indifference of many [19,34,35]. The fact is that blood is an invaluable asset that can save the life of a fellow human being. It is the highest act of generosity and selfless love, yet this lack of awareness emphasizes and underlines many of contemporary society’s issues. Prominent levels of awareness, a good attitude, and a fervent desire to donate blood should be utilized to emphasize the need of educating younger generations about the life-saving benefits of blood donation and providing them with accurate information about the overall requirements for blood donation [14,19,34,35,36]. Altruism, personal esteem needs, peer pressure and a sense of societal responsibility are among the primary motives for blood donation. [19,35]. The ethical gratification of blood donors for their contributions to therapeutic treatment of patients who require a blood transfusion and its derivatives can be a driving force for future donations as well as coverage for the blood donors and their families for blood and derivative needs [26,34,35,37]. Additional factors that can negatively affect behavioral intention include the lack of awareness, indifference as well as the ignorance of certain other aspects of the donation procedure. Yet, most individuals recognized the value of blood donation and were eager to donate blood, provided that the necessary conditions were met. Blood donation can be a stressful experience for novice blood donors, which may also explain their lower levels of satisfaction [38,39,40]. Individuals who are satisfied with their blood donation experience are more inclined to become regular blood donors, as donors tend to showcase greater confidence and trust regarding the blood donation process than non-donors [19,35,39,40].

#### 2.1.1. Altruistic and Egoistic Motives in Blood Donors

Advertising strategies are developed and coordinated by blood donation services, with the objective of not only recruiting new donors but also of retaining active blood donors [41,42]. In these cases, advertising emphasizes donating as an altruistic act and its societal advantages. Donors desire the personal gratification and benefits that come with giving blood. Benevolence is a philanthropic viewpoint in which both the donor and the receiver benefit [42,43,44,45]. Regardless of the behavioral factors that may lead to blood donation, the successful recruitment of potential donors necessitates a time-consuming and complex procedure.

Studies have revealed that altruism, social responsibility, and peer influence were the most stated reasons and motives behind blood donations [13,17,41]. The most significant reasons for continuing to donate blood after the first time were altruism and social responsibility [32,46,47]. People’s altruistic disposition can prove to be a crucial incentive for donating blood, as it meets their general desire to provide to their fellow human beings and promotes the solidarity contribution to societal well-being despite the fact that there is no evident personal gain [25,36,45,48]. As previously mentioned, satisfaction could be viewed as a consequence of an individual’s positive and altruistic attitude towards blood donation, and it is a primary motivator for a blood donor [36,49]. Piliavin and Callero [25] provide a comprehensive review of the relevant literature, in which it became apparent that despite altruism being the most frequently cited rationale that motivates an individual to donate blood, there are disparities amongst the other motivating factors for blood donation.

The safest blood donors are unpaid volunteers who contribute blood periodically. Individuals who actively donate blood without seeking a monetary reward are unlikely to have any incentive to conceal facts about their health and lifestyle that could preclude them from supplying blood, either temporarily or permanently, according to research from different countries [19,26,34,35]. Their fundamental intention is to benefit others as opposed to gain personal profit, with the gratification of knowing that they have contributed to the saving of a human life, a concept that aligns with the ideals of altruism and altruistic practices. Blood donation is a rudimentary act of unselfishness, distinguished by the deliberate act of offering services to anonymous receivers, emphasizing the selfless desire to benefit others, even at the expense of one’s own life [6,36,50,51]. Altruistic beliefs accentuate the notion of aiding others via donating. Donor intentions and behavioral changes can prove to be powerful driving forces in predicting potential blood donation behavior [14,33,52].

As external rewards for blood donation, motivations should raise the perceived value of the exchange and enhance the likelihood of a person donating blood [1,9,53]. Yet, it is unclear to what extent these motivations eventually entice individuals and facilitate the process, and even solidify it as a customary practice, to donate blood [9,53]. Although blood donation and other forms of volunteering are altruistic, even altruistic behaviors can have a selfish drive since they alter people’s socioeconomic status. This perception should be considered when developing new blood donation campaigns, as they should not only promote the humanitarian aspect of blood donation but also emphasize generosity and charitable ideals [32,50]. An alternative case is that people perceive blood donation as a personal reward, and these incentives do not occur as a result of third-party inducement. In contrast to charity, it is a paradigm in which the contributor reaps the rewards of their actions as lucrative or personally gratifying [45,47,54]. Altruism is a non-selfish act that enhances the prevalence of the group at some cost to the individual, whereas selfishness promotes the prevalence of individualism at some expense to the social collective [36,45,54]. Selfish and personal objectives might serve as the driving force behind volunteering and helping behavior. Donors report favorable benefits and an increase in pleasant mood following donation [46,47,48,50]. This theory originates from the basic principle of mutual cooperation and is motivated by self-interest motives [55].

Individuals who provide blood for payment are typically motivated by what they will receive in exchange for their blood, not by a desire to help others. Additionally, independent of age, education, or money, only social responsibility had a substantial influence on persons’ willingness to donate blood [9,12,14]. As a result, the urge to give blood is related with a mix of self-assurance and self-esteem motives, as well as a high level of altruistic motivation [2,45,46]. Ferguson, Farrell and Lawrence [32] claim that attitudes and views centered on personal rather than social value predicted future blood donations. When blood donors were subjected to messages of generosity and compassion rather than selflessness and self-sacrifice, they were more likely to donate blood. In the case of committed blood donors, significant attitudinal changes to donate blood were detected between benevolent and altruistic messages.

#### 2.1.2. Attitudinal Differences between Repeat Blood Donors and Non-Donors

Volunteerism incorporates the principles of prosocial behavior and altruism, with prosocial behavior relating to engagement and goal setting and altruism to the motivation to participate. Numerous studies argue that volunteering is connected to altruistic personality and traits such as self-esteem, generosity, extroversion, and a strong sense of obligation to others [2,56,57]. Most definitions of altruism have emphasized the value of free choice while focusing on helping “others” without pecuniary benefit. The nature and expression of altruism in voluntary activities is defined and influenced by how others react to these circumstances. Occasional volunteering is motivated by extreme events of need, whereas systematic volunteering is motivated by people’s personalities, values, socialization, and decision making for voluntary action [25,48]. Although altruism appears to be the most significant motive for becoming and remaining a volunteer blood donor, except in emergency situations, the rate of voluntary blood donors in most countries is insufficient to cover transfusion demands [11,14,57].

A favorable attitude towards donation, subjective norms, awareness, and confidence in a successful and safe donation all increase the likelihood of adopting blood donation behavior in donors [14,57,58,59]. Assisting those in need is a natural human instinct. Volunteer services and activities enable people to externalize their sentiments of solidarity by assuming the role of a helper or provider. The type of assistance supplied is a crucial consideration since it entails changing factors, motivations, and personality attributes each time. Individuals’ own values and sociability, in particular, have a significant effect on systematic volunteering [13,14,59,60]. Blood donation volunteering demands commitment and responsibility, alongside other qualities that are not inherent in all volunteer efforts. In some cases, blood donation services attract people who value their safety, lack empathy, or have narcistic and egotistical intentions [9,57,59]. This finding has prompted many scholars to consider volunteering as a concept that is not based on purely humanitarian and altruistic motives but that is tied to selfish motives, such as gaining a competitive edge when applying for admission to institutions or claiming job positions.

A common research topic is the effectiveness of financial and non-financial inducements made available to individuals to improve the attractiveness and engagement of blood donors. Current studies have indicated that while specific incentives may induce blood donation for particular categories of people, keeping them as regular donors is more challenging [9,19,39]. Although it is acknowledged that in many cultures, referring to blood donors as “customers” is vulgar and unethical, the art of successful blood donor recruitment has many parallels to what marketing research would term customer service [9,13]. The incentives for voluntary blood donation must be humanitarian primarily, but the recruitment of blood donors should involve innovative tactics to recruit necessary new blood donors. Compelling advertising needs to emphasize the altruistic nature of voluntary blood donation and instill a sense of social responsibility [41,50,58]. The gesture of voluntary blood donation acknowledges the aspects of contribution and charitable giving instead of just receiving material compensation. Understanding the motives that encourage individuals to donate blood without financial remuneration is critical for increasing the success of programs designed to recruit new blood donors and maintain existing ones [42,61,62].

Non-donors do not give blood as they have never been persuaded to act so, indicating that their decision is not affected by external causes. Attitudes also guide an individual’s approach in all social institutions [34,39,63]. Social phenomena, or how people perceive and respond to the social aspects in their surrounding environments, can be influenced by a variety of psychological and social occurrences [34,39,63]. Hence, in the case of blood donation behavior, the ultimate decision is entirely personal and is made after internal motivation has been solidified after processing perceptions and evaluating several considerations [57,62]. The emotional state before donating blood is especially relevant among young blood donors, influencing future behavior [57,62].

#### 2.1.3. Personality Traits

Our preferences and behavior are heavily influenced by our personality, what type of lifestyle we decide upon, the beliefs we uphold, and the influences we receive from our external environment. Decisions are often influenced by different indications received from our social environment as well as the marketing environment via advertising [64,65,66]. Furthermore, personality is people’s accumulation of internal traits that govern how they behave, their decision-making processes, and preferences, which can be classified into separate categories based on its distinctive characteristics [64,65,66].

The HEXACO personality model is widely acknowledged and utilized in research and practice areas such as personal development, job selection, and counseling [67,68]. This model identifies six fundamental personality dimensions: Honesty–Humility, Emotionality, eXtraversion, Agreeableness, Conscientiousness, and Openness to Experience. Each individual has a distinct combination of these traits that distinguishes them. However, it should be noted that personality traits are not the sole element determining people’s behavior or decisions. Other elements, such as culture, environment, upbringing, and experiences, can also influence a person’s behavior and decisions. This suggests that personality is a multifaceted phenomenon involving the interplay of several elements in detail [64,65,66,67].

The Honesty–Humility trait relates to a person’s truthfulness, compassion, and integrity when interacting with others. High scores imply honesty, modesty, and unassuming habits, whereas low levels reflect arrogance and haughtiness. A high score of eXtraversion indicates that a person is outgoing, sociable, and energetic, whereas a low score indicates someone who is reserved, introverted, and timid. Emotionality relates to emotional steadiness. High scores suggest emotional stability, calmness, and confidence, whereas low values indicate emotional instability, anxiety, and sensitivity. Agreeableness refers to cooperation and amiability. High scores show friendliness, kindness, and compassion, whereas low levels indicate coldness, remoteness, and a lack of sympathy. Conscientiousness is a trait that describes a sense of duty and self-discipline. High scores reflect a person’s organization, dependability, and goal orientation, whilst low levels represent irresponsibility and a lack of organization. The Openness to Experience attribute describes receptivity and curiosity concerning new experiences. A person with a high score is open-minded, creative, and curious, whereas a person with a low score is closed-minded and conventional [67,68,69].

Acknowledging the vital role of personality traits, we attempted to include this dimension in our research by incorporating the HEXACO personality model, one of the most well-known and researched models. In its full version, it consists of 100 items in the form of statements that the respondent is asked to declare the degree of agreement (1–5 scale), but is also available in shorter versions (60 and 24 items). To our knowledge, no prior research has been conducted utilizing the 24-item HEXACO personality model [70] in the context of blood donation behavior. Having said that, HEXACO has been the main point of interest in related research areas concentrating mostly on the altruistic and pro-social features of personality traits [69,71,72]. Thus, it is deemed necessary to investigate how personality theories operate in terms of people’s altruistic and selfish tendencies, particularly in connection with voluntary blood donation. This way, we can approach our results with greater precision and reliability in terms of the search for the traits that we anticipate demonstrating associations with our findings. Regardless of the structural differences and similarities displayed by the personality models, we are interested in the traits connected with the case of voluntary blood donation, since it concerns a dimension that addresses the altruistic and prosocial nature of individuals.

The Big Five Factor Model (BFM), also known as OCEAN, is an internationally recognized personality framework that is commonly employed in personality assessments [73,74,75,76]. It comprises 44 items in the form of statements to which the respondent is asked to declare their degree of agreement (1–5 scale). It has received widespread recognition as a trustworthy instrument for exploring unique characteristics in a variety of situations, including medicinal, counseling, and industrial settings. This model is based on current personality questionnaire forms and is derived from lexical analysis and empirical studies. This personality model delineates the unique patterns according to how individuals process ideas, sentiments, and behaviors, condensing these in five overarching dimensions: Neuroticism, Extraversion, Openness, Agreeableness, and Conscientiousness [73,74,75]. We made reference to the Five Factor model, where we searched for those traits that fit within the Big Five model and yet can be associated with the personality traits of HEXACO, as the literature explicitly associating HEXACO with voluntary blood donation behavior is limited and inadequate. In this instance, the BFM’s Agreeableness, Openness to Experience, and low levels of Neuroticism, which is adversely connected with prosocial and altruistic behaviors, are appropriate attributes to evaluate in our research and construct legitimate associations with HEXACO’s traits [69,71,72,77]. According to Ashton and Lee’s numerous studies [67,68,69,71], the altruistic element in HEXACO is primarily an amalgam of the traits Honesty–Humility, Emotionality, and Agreeableness, as described by the authors: “*the overall tendency to be altruistic or to be antagonistic will represent a blend of those three dimensions*” [69]. Should individuals exhibit prominent levels in these attributes, they are prone to engage in altruistic behaviors, selflessness, empathy, or even develop a keen sense of personal and societal responsibility. These traits, which are anticipated to be revealed as expected outcomes in the scope of our research, play a critical role in determining the degree to which people assessed with the HEXACO model exhibit tendencies towards altruistic behaviors.

In studies of blood and organ donation behavior, social frameworks have underlined the devotion to fellow humans and empathy as deciding variables for determining the meaning of positive social behaviors and developed conceptual frameworks to explain the reasoning behind blood and organ donation intention [76,78,79,80]. Based on this idea, when a person perceives one’s suffering or hardships, their tendency regarding altruism is reinforced. Nevertheless, in cases where it satisfies an individual’s criteria, an egoistic mentality can be a motivator for prosocial behavior. Although excessive egotism could contradict one’s ambitions, it can potentially be advantageous in certain circumstances when combined with voluntary ideals and a sense of one’s own involvement in tackling society barriers [47,81]. In its broadest sense, the Agreeableness trait encompasses acquaintances who endorse altruistic dispositions by engaging in emotional support endeavors and are interested in care for other human beings. These individuals are described as compassionate, charitable, and sensitive, with an inherent tendency to actively participate to social events through volunteer initiatives while demonstrating satisfaction in complying with societal norms for the purpose of the communal good. Those with low scores, on the other hand, exhibit self-centered behaviors regardless of whether there is personal profit, which typically translates into antagonism. Despite their cynicism and competitiveness, which may lead to disagreeable and unpleasant behavior, they are more inclined to participate in prosocial activities such as blood donation to benefit the welfare of others. Those with high Agreeableness may be more cognizant of the value of blood donation in saving lives and improving the health of others due to their empathic nature. This trait serves a critical role in encouraging philanthropic giving and highlighting the possibility to actively contribute to the welfare and benefit of society [74,76,78,82,83]. The Neuroticism trait indicates individual variances caused by negative emotions such as anxiety, melancholy, or disgust, as well as how this tension comes across cognitively and behaviorally. A low ranking in this trait does not always indicate the existence of ideal psychological health but rather a calm and self-assured demeanor. Such personalities exhibit emotional stability, which makes them less susceptible to anxieties or other stressful situations, but it mostly indicates strong mental endurance. In challenging times, they maintain their tempers and remain optimistic about the future. At the other end of the spectrum, their oversensitivity to negative emotions can cause abrupt changes in behavior and mood, generating instability in their interpersonal connections and their view of their social environment [82,84,85]. Openness to Experience is a trait connected with artistic sensitivity, the need for adventure, and an appetite for creativity and originality. Inventiveness, curiosity, and openness to new experiences are all examples of this characteristic. This personality trait is observed in people who have a creative tendency, unconventional beliefs, an acute emotional sensibility, and an attraction to the arts. A low score on this factor, on the other hand, demonstrates individuals who share more conventional beliefs, tend towards conservatism, and are reluctant to change [74,82,83,85].

### 2.2. Emotional Arousal in the Context of Blood Donation

As aforementioned, the reasons for being a blood donor derive from either internal incentives such as compassion and personal commitment, or external motivations such as remuneration and a sense of recognition. To ensure the satisfaction of potential blood donors, a blood donation service must develop and reflect the sentiment of devotion. Since the requirements and the motives for donating blood alter and change with time, blood donors should not be taken for granted. It is of crucial significance to devise particular tactics for rewarding and acknowledging the offer of voluntary blood donors.

Due to the delicate balance between blood supply and demand, the various qualified blood suppliers are continuously looking for more efficient ways to recruit blood donors [38,86]. The profile of blood donors is necessary and essential information in the development of strategies to attract and retain current ones. The objective is to acquire a deeper understanding of the characteristics of blood donors and to develop more successful campaigns [9,10,59]. In the context of the ever-increasing need to maintain adequate quantities of blood, governments around the world, health authorities, and non-governmental organizations, as well as blood banks and transfusion centers and services, are implementing marketing strategies to attract voluntary blood donors and maintaining them so that they become regular blood donors [10,41,87]. In this endeavor, social marketing plays a key role in emphasizing the numerous benefits of blood donation, both on an individual as well as a social scale [21,41,42]. In contrast to traditional marketing ideas, social marketing focuses on citizen behavioral change through strategies that address social issues and seek to achieve social change. It aims to achieve the adoption of innovative ideas and attitudes by the community and the alteration of established circumstances, attitudes or even actions with the common aspect of societal improvement and wealth [41,42,85]. Besides that, social marketing attempts to alter societal structures to support individual change.

In reality, transfusion centers and blood banks are gradually recognizing and acknowledging the necessity and influence of social marketing on recruiting donors and reinforcing their trust to ensure a steady supply of blood donations [10,41,42]. Social marketing experts should provide their products or services in conjunction with a social marketing campaign that is centered on the advantages of the targeted audience. Prior to this, marketing research is required to understand the audience’s interests, requirements, values, and motivation in hope of maximizing the potential advertising and emotional appeals in every relevant case [10,13,41]. As previously discussed, it is critical to comprehend the factors and attitudes that influence and inspire volunteers. Understanding how external and internal attitudes differentiate and translate in attitudinal and behavioral changes is necessary to develop and implement alternative approaches in practical marketing techniques, as advertising messages are perceived differently depending on the audience to whom they are directed. Similarly, Sundermann, Boenigk and Willems [61] underline the need of market segmentation, targeting specific groups, and the implementation of personalized marketing, depending on the market segment you are dealing with in attempting to approach blood donation. One of the most effective strategies available to advertisers in campaign planning is the recruitment of blood donor volunteers by appealing to people’s altruistic ideals. Donating blood is regarded as an archetypal, philanthropic act, and altruism is among the most commonly self-reported motives for donating blood. As a result, the approach of attracting newcomers should be designed to increase their preparedness for future donations [8,9,10]. Long-term and sessional donors both have significant altruistic motives for blood donation, whereas non-blood donors are more likely to be motivated by reasons relating to self-esteem and confidence than regular blood donors. Therefore, it appears that the habit of frequent blood donation is not solely associated with other humanitarian behaviors or a specific set of incentives [8,9,10]. Blood donation organizations throughout the world are striving for compelling incentives to boost the success of blood donor attraction and retention campaigns, along with the increasing regularity of blood donation [9,53,88].

While negative attitudes toward blood donation have not been documented, a sizable portion of the general population is hesitant to donate blood [2,12,13]. According to most research, the fear of pain and/or infection, personal phobias (a fear of needles, dizziness, etc.) are also identified as reasons inhibiting blood donation. Illness, the incidence of low hematocrit or some kind of anemia, a lack of time, the occurrence of post-donation symptoms such as dizziness and headache, and unpleasant medical and nursing staff at blood transfusion facilities are all significant deterrents [62,89,90]. Additionally, some people suggested they have not donated blood due to the ineffective efforts to attract donors by blood donation services as potential donors do not know where to seek information or because the nearest blood collection facility is a considerable distance away, but they also claim they never started the procedure because there was no need to for a relative or friend [33,37,50]. Furthermore, individuals have indicated that poor service from the personnel and different organizational challenges are disincentives for blood donors. Anxiety and uneasiness, which many blood donors experience before beginning the blood collection process, a previous unpleasant experience and fear for the safety of the overall procedure have a comparable impact [42,62,86,90]. First-time blood donors are more anxious and do not “feel comfortable” with the entire procedure, owing to inexperience or a lack of information compared to frequent blood donors, who are more acquainted and experienced [91].

Several studies have investigated the dynamics of emotional and rational appeals in social cause advertising, shedding light on their effectiveness to provoke behavioral changes. Casais and Pereira [92] found that, contrary to theoretical expectations, advertisements for voluntary blood donations were largely categorized as rational, emphasizing the effectiveness of rational appeals in social media campaigns. Another study focused on threat as a message appeal in social cause advertising during the COVID-19 pandemic, revealing the impact of agency and communal orientations on responses to threat-inclusive advertisements [93]. This survey-based research highlighted that individuals characterized by these orientations might be more susceptible to messages containing threat appeals in social cause ads. Meanwhile, Gomes and Casais [94] explored the effects of threat appeals in campaigns related to anorexia nervosa across social media platforms. Utilizing sentiment analysis, they discovered a mix of emotions, both positive (such as support and compassion) and negative (including fear and sadness), triggered by these campaigns. Their analysis revealed the limitations of relying solely on emojis to understand emotional responses compared to text analysis, underlining the complex range of emotions elicited by threat-based social media campaigns. Casais and Proença [95] conducted a content analysis of 375 HIV/AIDS prevention TV ads across four European countries, emphasizing the use of positive and negative appeals in social advertising. They found that positive appeals were prevalent, particularly in rhetoric, music, and voice tonality, while negative appeals were more prominent in narratives and visual elements. Negative appeals were more common in countries with higher uncertainty avoidance indexes and epidemic incidence rates. Meanwhile, an investigation on blood donation intention compared factual and attitudinal approaches [96]. Factual questions about donors’ history and social associations and attitudinal inquiries adapted from the TPB framework showed similar predictive validity, with factual questions being easier to implement. Ultimately, the authors stressed the challenge in predicting and distinguishing behavioral intentions in blood donation. These studies collectively shed light on the significance of appeals in social advertising, offering frameworks and insights for categorizing health-focused ads and exploring different approaches to predict behavioral intentions. They emphasize the significance of understanding emotional and rational appeals, threat inclusivity, and the nuances of emotional responses in crafting effective social marketing campaigns. They shed light on the significance of emotional appeals in social advertising, offering frameworks and insights for exploring different approaches to predict behavioral intentions.

Contemporary research has provided valuable insights into the factors influencing blood donation intentions and behaviors across different contexts. Matubatuba et al. [97] pointed out three major factors that have the highest effects in determining intentions of blood donations among South African consumers, which include an awareness of consequences, the ascription of responsibility and personal norms. Blood donations in neighborhood settings are influenced by social contagion, according to Schröder et al. [98], with donor couples showing a greater positive effect. To maintain attitudinal loyalty among active donors, Robaina-Calderín et al. [99] identified motivations, obstacles and service quality as factors influencing performing SEM analysis that revealed the need for customized strategies for donor retention. Mohanty et al. [100] examined message framing and perceived risk effects on donation intention, proving positivity works best under certain risk conditions, while negativity works where risks are perceived to be high. Gong et al. [101] investigated the effectiveness of incentives, finding that eligibility for free blood transfusions is more effective than improving credit scores, primarily due to the perceived attractiveness and threat to freedom. Emphasizing the emotional rewards and family incentives of blood donation, Silva Carlos and Rodrigues [102] also identified obstacles like needle phobia and the lack of knowledge about what motivates blood donors. Lastly, Liu and Han [103], on a sample of Chinese university students, applied the theory of planned behavior that showed attitudes, subjective norms, and self-efficacy to be significant predictors of blood donation intentions, with attitudes mediating the effects of altruism and social norms. Collectively, these studies highlight the complex nature of blood donation behavior as well as its implications for donor recruitment efforts in terms of targeted strategies.

The literature on blood donation behavior highlights various motivational factors, including altruistic and egoistic incentives. Previous studies have shown that emotional appeals in advertising can significantly impact individuals’ intentions to donate blood [81,85,99,101,102]. Additionally, personality traits, such as those outlined in the HEXACO model, have been linked to prosocial behaviors, suggesting that traits like Honesty–Humility, Emotionality, and Agreeableness may play crucial roles in influencing donation intentions. Based on these insights, we propose the following hypotheses:

**Hypothesis 1 (H1).**  *HEXACO’s Honesty–Humility (HH) personality trait directly influences the behavioral intention to donate (BI).*

**Hypothesis 2 (H2).**  *HEXACO’s Emotionality (E) personality trait directly influences the behavioral intention to donate (BI).*

**Hypothesis 3 (H3).**  *HEXACO’s Agreeableness (A) personality trait directly influences the behavioral intention to donate (BI).*

**Hypothesis 4a (H4a).**  *Attitudes towards the advertisement (ADDs) have a direct effect on DESPositive (DESPos).*

**Hypothesis 4b (H4b).**  *Attitudes towards the advertisement (ADDs) have a direct effect on DESNegative (DESNeg).*

**Hypothesis 5a (H5a).**  *DESPositive (DESPos) directly influences the behavioral intention to donate (BI).*

**Hypothesis 5b (H5b).**  *DESNegative (DESNeg) directly influences the behavioral intention to donate (BI).*

**Hypothesis 6a (H6a).**  *The link between HEXACO’s Honesty–Humility (HH) personality trait and the behavioral intention to donate (BI) is mediated by attitudes towards the advertisement (ADD) and the emotional arousals (DESPos and DESNeg).*

**Hypothesis 6b (H6b).**  *The link between HEXACO’s Emotionality (E) personality trait and the behavioral intention to donate (BI) is mediated by attitudes towards the advertisement (ADDs) and the emotional arousals (DESPos and DESNeg).*

**Hypothesis 6c (H6c).**  *The link between HEXACO’s Agreeableness (A) personality trait and the behavioral intention to donate (BI) is mediated by attitudes towards the advertisement (ADDs) and the emotional arousals (DESPos and DESNeg).*

**Hypothesis 7 (H7).**  *The type of message (altruistic vs. egoistic) moderates the relationship between emotional arousal (DESPos and DESNeg) and the behavioral intention to donate (BI).*

## 3. Research Methodology

### 3.1. Experimental Procedure and Setup

**Figure 1:**
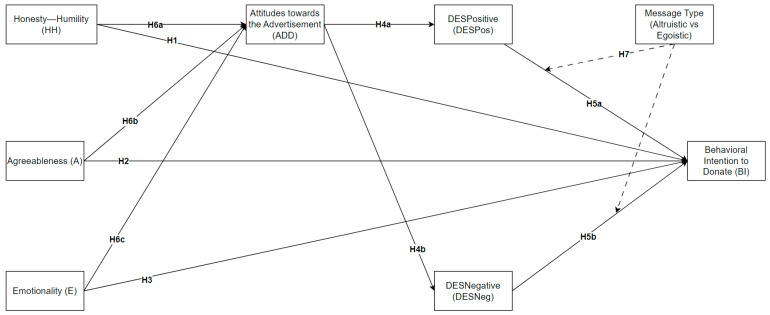
Conceptual research model.

The primary objective of this study was to identify and investigate behavioral intentions and attitudinal changes among individuals who could function as donors, specifically in the case of blood donation. The research aims to offer insight into the various aspects that influence people’s decision to donate blood, including the impact of emotional appeals, personality traits, and attitudes to blood donation advertisements. The present investigation was envisioned to acquire an in-depth understanding of the fundamental factors that drive individuals’ involvement in such initiatives by delving into the intricate interplay between psychological variables and individuals’ motivation to participate in the life-saving act of blood donation. This research approach uses quantitative data analysis methodologies to examine the dynamic interactions between specific factors, seeking to improve public health perspectives and practices related to voluntary blood donation. A visual layout of the specific research framework is available in Figure 1.

**Figure A1:**
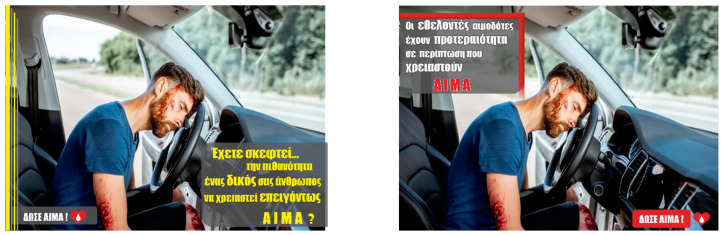
Variants of the fear ad. On the left, we showcase the ad with an altruistic message (Ad01a: “Have you thought about the possibility that one of your people urgently needs blood?”), and on the right, we display it with an egoistic message (Ad01e: “Volunteer blood donors have priority in case they need blood.”) [105].

We devised an experimental approach to investigate the reasons that lead to future voluntary behavior. Considering the influence of positive and negative emotional appeals triggered by advertisements for promoting blood donation, we collaborated with a professional graphics design firm to produce two sets of three advertisements (Ad01–Ad06). Each set of ads displayed a different emotion (positive and negative emotional appeal). Then, each of the ads was delivered in two versions, each one featuring a different textual message (altruistic and egoistic), ending up with a total of twelve (12) advertisements in a 2 × 2 experimental design (positive vs. negative emotional appeal; altruistic vs. egoistic message) as depicted in Table 1. We addressed altruistic and egoistic motivations and made deliberate efforts to incorporate these characteristics into messages aimed at persuading participants to donate blood. Consequently, each advertisement had two versions, one with an altruistic message and one with an egoistic message, allowing us to investigate the influence of these appeals on individuals’ behavioral intentions. Examples of altruistic messages are “Have you considered the possibility that one of your close relatives needs blood urgently?” (Figure A1) and “Is a small and quick pinch so important...that you refuse to save the lives of three people?”, and the corresponding egoistic messages are “Volunteer blood donors have priority in case they need blood” (Figure A1) and “Donating blood can increase the life span of the donor” [104,105].

An online pre-survey was carried out to confirm that the stimuli meant for the experimental study would effectively elicit the desired emotions. Utilizing a convenient sample of 66 participants, thirty images—fifteen positive and fifteen negative—were rated across six emotions, joy, inspiration, interest, guilt, disgust and fear, with each emotion consisting of five diverse pictures. Every image was assessed on a 5-point Likert scale so as to decide upon the intensity of emotion elicited. These findings assisted in selecting the six images which had the strongest emotional impact—three positive and three negative ones. Additionally, message framings aligning with the images’ themes, conveying either altruistic or egoistic intentions, were chosen. This pretest phase was designed to ensure that advertisements used in the main experiment resonated effectively with respondents and induced the anticipated emotions accordingly [105].

**Figure A2:**
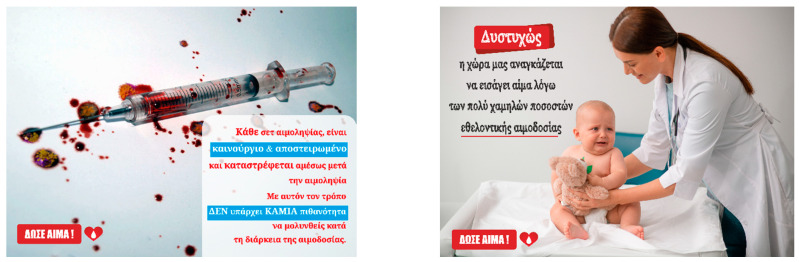
Illustrations of negative appeal ads. On the left, we showcase the disgust emotional appeal ad with egoistic message framing (Ad03e: “Each blood collection set is new and sterile, and is destroyed immediately after blood collection. This way there is NO chance of getting infected during the blood donation”), and on the right, we show the ad using guilt appeal with altruistic message framing (Ad02a: “Unfortunately, our country is forced to import blood due to the very low rates of voluntary blood donation”) [105].

**Figure A3:**
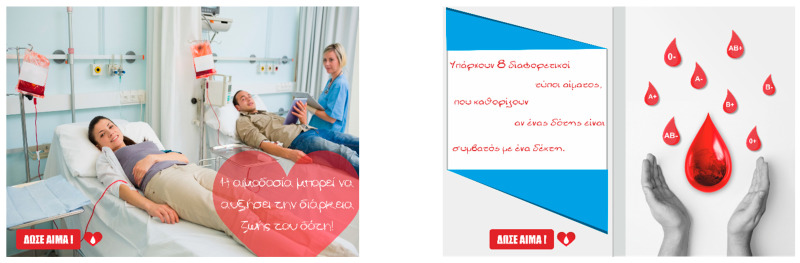
Illustrations of the positive appeal ads. On the left we, showcase the positive emotional arousal inspiration with egoistic message framing (Ad04e: “People who volunteer for humanitarian causes usually live longer”), and on the right, the interesting positive arousal with altruistic message framing is shown (Ad05a: “There are 8 different blood types that determine whether a donor is compatible with a recipient”) [105].

Figure A1 in Appendix A depicts the two versions of the stimulus used to evoke the negative emotion of fear (one with altruistic message framing and one with egoistic message framing). Additionally, Figure A2 and Figure A3 showcase examples of images for positive and negative emotions with their corresponding textual messages (altruistic or egoistic).

We placed particular emphasis on how diverse emotions lead to voluntary blood donation. The advertisements were constructed in such a manner that we were able to convey distinct emotions while keeping the other criteria identical. We concentrated on interest, inspiration, and joy for the positive emotions, while the negative emotions were designed to instill guilt, fear, and disgust to participants. We avoided utilizing adverts from current existing volunteer blood donation services (for example, the Red Cross) to prevent any preconceptions and favoritism towards these organizations, which has been shown to influence donors’ behavioral intentions [8,10,41].

To ensure a thorough and rigorous analysis, a quantitative non-experimental correlational 2 × 2 experiment was conducted, employing a repeated-measures within-subject design. A quantitative non-experimental correlational research design was chosen as it offers empirical validation and statistical evidence to support the proposed theoretical framework, whilst its widespread applicability allows for subsequent research endeavors [106,107]. The primary aim of this methodology was to elucidate the factors affecting users’ intentions towards blood donation intentions. Data collection was conducted through a structured online questionnaire, which was self-administered, encompassing both existing and potential blood donors. This design choice involved assigning the same participants to all treatment conditions, enabling a direct comparison of their responses across the different variables [108,109]. To minimize potential user bias, a “*completely randomized factorial design was employed for randomization to assign participants to all treatment conditions*” [110,111]. The default condition for each participant was determined by a random order selection process.

Data collection was performed through an electronic questionnaire in Greece, using a distribution tactic that included both academic institutions and the wider social fabric. This effort was organized to capture the broad spectrum of the Greek population and to provide an analytical overview of the main trends in voluntary blood donation from the perspective of our research. The questionnaire consisted of 38 items (including all items from HEXACO-24) and was posted on various social media platforms with the aim of enhancing the participation of people from different socioeconomic backgrounds, thereby promoting diversity in our sample.

Participation was entirely voluntary. The experiment took place between 20 October 2022 and 24 December 2022 at the Department of Management Science and Technology of the University of Patras, Greece. The participants in the study were given an overview of the study’s process and goals, as well as a brief explanation of voluntarism and blood donation principles. They were additionally informed that they could withdraw from the experiment at any point in the process and for any reason, as well as being provided with information on how the data would be collected and analyzed. They were additionally informed of the university’s rigorous commitment to data protection standards, which ensured the security and safety of their personal information during the study. Finally, participants were requested to read and sign a participant consent form. Following that, participants completed a pre-test questionnaire to acquire information about their demographics (age, gender, education, etc.) and voluntary activities. After the completion of the questionnaire, participants were shown the 12 advertisements as static images on a computer screen in random order. In order to increase survey participation, various sample development and collection techniques were adopted, such as the snowball sampling method [112], which encouraged respondents to forward the questionnaire to their personal contacts (friends, family, etc.) upon the completion of it. In addition, as an incentive for further participation, the opportunity to participate in a raffle with EUR one hundred gift vouchers was offered in order to motivate the participants upon completing the questionnaire.

### 3.2. Instruments and Metrics

Following exposure to the advertisements, the experimental procedure was terminated, and individuals were prompted to complete a post-test questionnaire. The post-test questionnaire was designed to investigate and evaluate the emotional appeal in each case, the attitudes of participants towards the advertisements, and their future behavioral intention to donate blood voluntarily. To measure the emotional arousal, we implemented the Greek version of the Differential Emotions Scale (DES). The construct included items to measure positive emotions (joy, inspiration, and interest), namely DESPositive, and negative emotions (guilt, disgust, and fear), namely DESNegative. This was decided to confirm that we achieved our aim in terms of the depicted emotion in each respective case [113]. Attitude towards the ad was measured with a five-item semantic differential scale obtained and adapted from Holbrook and Batra [114] and Ranganathan and Henley [115]. The construct included the items of likeness (dislike/like the ad), favorable reaction (unfavorable/unfavorable), feelings towards the ad (negative/positive), overall attitude (bad/good) and information (uninformative/informative). Behavioral intention to donate blood was measured with a three-item scale adapted from [116]. Finally, personality traits were measured in six dimensions using the well-established HEXACO-24 Personality Inventory Revised [70] to measure Honesty–Humility, Emotionality, Extraversion, Conscientiousness, Agreeableness and Openness to Experience. All items are depicted in Appendix A. As explained in detail in the literature section, the altruistic element in HEXACO is primarily a combination of the traits Honesty–Humility, Emotionality, and Agreeableness; thus, in our endeavor, we placed particular emphasis only on those personality traits that were most related to the scope of our research. Future research will address the aspects of how the other personality traits could potentially affect the outcome of our research model.

The evaluation of the variables was carried out using a five-point Likert scale. This scale underwent careful modifications and translations in order to faithfully preserve the meaning of the questions and guarantee the statistical validity of the research. For the accurate translation of the questions, so that they could be understood by the Greek public, this responsibility was taken by a specialized multilingual scientific consultant. In addition, a preliminary inspection was performed to identify and correct possible wording inconsistencies, while the data from the pilot test were excluded from the final processing.

### 3.3. Sample Profile

As illustrated in Table 2, the descriptive statistics of the sample comprising 462 participants reflect an equal gender distribution, with females accounting for 51.9% of respondents and males accounting for 48.1%. In terms of age groups, they range from 18 to 60+ years, with the bulk of respondents being 18–25 years old (140 participants, 30.3%) and the smallest group over 60 years old (9 participants, 1.9%). As far as the educational background is concerned, the sample varies from high school graduates to those holding a doctoral degree, with the largest proportion being undergraduate students (165 participants, 35.7%) and the smallest proportion being those with a doctoral degree (4 participants, 0.9%).

The decision to focus the study on the young adults age group is grounded on numerous arguments. Age influences voluntary blood donation as an individual’s physical condition could deteriorate with time, making the process more difficult or gradually decreasing the frequency with which voluntary blood donations are carried out [19,20,35]. A phenomenon that correlates with health problems that are frequently connected with aging may pose a challenge. Furthermore, with age, responsibilities and obligations escalate. Overall, it has been observed that frequent blood donation volunteers tend to be younger in age [19,20,117]. From the perspective of choosing the main audience for targeting blood donation campaigns, young people also feature as the group with the better potential to offer blood with a higher frequency and for a longer period [14,19,20,55,117].

## 4. Data Analysis and Results

In the present investigation, the use of Smart-PLS4 software (version 4.1.0.0) for data analysis was utilized, wherein the structural equation modeling (SEM) was deployed as a key methodological instrument. The technique is well acknowledged for its efficacy in performing variance-based SEM analyses in the field of management and social sciences as indicated by Nitzl et al. [118]. Moreover, PLS-SEM was incorporated in this study as it examines causal modeling and aims at maximizing the amount of variance explained in dependent latent constructs. Therefore, with regard to beta coefficient accuracy as well as standard errors and reliability metrics, this research followed Wong’s [119] methodological guidelines. In line with the reflective measurement model assessment criteria, every indicator needed to be rightly associated with its corresponding latent construct and have outer loadings greater than 0.7, which is mandatory from an evaluative perspective.

### 4.1. Common Method Bias

In order to ensure the validity and accuracy of our study, we conducted a comprehensive assessment for common method bias (CMB), following established methodological guidelines by Podsakoff et al. [120]. Harman’s single-factor test was performed to control CMB and to examine whether a single factor could explain most of our model’s variance [120]. The results of unrotated Principal Factor Analysis indicated that the general factor explained 22.067% of the total variance, which is fairly below the critical value and threshold of 50%. Thus, CMB was not a problem in our study.

### 4.2. Measurement Model

At the onset of using Partial Least Squares Structural Equation Modeling (PLS-SEM), a thorough measurement model is assessed. As part of this procedure, reflective indicators are used to quantitatively evaluate the constructs in the model. The evaluation includes measures like composite reliability, indicator reliability, convergent validity and discriminant validity as prescribed by Hair et al. [121]. Indicator reliability is the basic step in examining the measurement model, where its purpose is to determine how much variance in a variable can be explained by the construct that it is intended to measure, as stated according to Chin [122]. These measurements become important through large magnitudes of outer loadings, as described by Wong [119], which should ideally be more than 0.70, with reference from Chin [123]. However, Vinzi et al. [124] recently noted that factor loading above 0.7 was preferred, but social science research has numerous examples where outer loadings were below this level. Decisions on whether or not to delete items with low loadings must include a careful consideration of their contribution to both composite reliability and convergent validity so that precipitous removal can be avoided. Indicators exhibiting outer loadings in the range of 0.40 to 0.70 may be considered for removal only if such action significantly contributes to enhancing the construct’s composite reliability or its average variance extracted (AVE), thus meeting the standards set forth by Hair et al. [125].

As evidenced in Table 3, the optimization of the measurement model led to the exclusion of one indicator (AAD5) due to their substandard factor loadings (<0.500), adhering to the guidelines proposed by Gefen and Straub [126].

The evaluation of reliability in this study was carried out by using Cronbach’s alpha, rho_A and composite reliability as the main metrics of assessment. Moreover, AAD, BI, DESNEG, DESPOS and E measures were higher than the minimum threshold of 0.700 established by Wasko and Faraj [127], whilst the rest exhibited moderate to high reliability based on [128,129,130]. The rho_A statistic that is conceptually placed between Cronbach’s alpha and composite reliability, which follows Sarstedt et al.’s [131] argument, also outperformed the 0.7 cut-off point in most cases, indicating strong support for Henseler et al.’s [132] findings about reliability. Furthermore, convergent validity was regarded as acceptable, given that average variance extracted (AVE) was greater than 0.50 for most constructs, which is consistent with Fornell and Larcker’s [133] recommendations. Nevertheless, as posited by Fornell and Larcker [133], the construct retains sufficient convergent validity if the average variance extracted (AVE) falls below 0.5, provided that the composite reliability exceeds 0.6. Discriminant validity was assessed using inter-construct correlations against the square root of AVE as outlined by Fornell and Larcker [133], while the heterotrait–monotrait correlation ratio (HTMT), introduced by Henseler, Hubona and Ray [132], was also used. It can be seen from Table 4 and Table 5 that all derived values were below the strict limit of 0.85, evidencing discriminant validity.

### 4.3. Structural Model

The analysis of the structural model within the stated research model was carried out by assessing R^2^ values, Q^2^ values and path coefficients’ significance levels. In this study, R^2^ values varied, ranging from 0.356 for behavioral intention to donate to 0.132 for DESNegative, 0.272 for DESPositive, and finally, 0.083 for AAD, thus confirming that they fell within the expected range of 0 to 1. This model’s predictability was checked through Q^2^ numbers, which were as follows: Behavioral Intention to Donate—0.021, DESNegative—0.024, DESPositive—0.024 and AAD—0.060. The hypothesis testing procedure demonstrated the reliability of the model since it asserted the significance of the interrelations amongst constructs. Path coefficients were tested using the bootstrapping method as per Sarstedt, Ringle and Hair’s [131] protocols. Additionally, Preacher and Hayes’s [134] mediation analysis methods were used for this research, which also employed Streukens and Leroi-Werelds’s [135] recommended bias-corrected, one-tailed bootstrap sample size of 10,000. The results obtained from these analytical procedures can be seen in Table 6 below.

The results revealed a significant influence of Honesty–Humility (HH) on behavioral intention to donate (BI) (β = 0.087, *t* = 2.186, *p* < 0.05), lending support to H1. Conversely, Emotionality (E) did not significantly affect behavioral intention to donate (BI) (β = 0.018, *t* = 0.435, *p* > 0.05), leading to the rejection of H2. Similarly, Agreeableness (A) also failed to show a significant impact on behavioral intention to donate (BI) (β = 0.033, *t* = 0.811, *p* > 0.05), resulting in the rejection of H3. Additionally, attitudes towards advertisement (AADs) had a significant positive effect on negative emotional arousal (DESNEG) (β = 0.363, *t* = 8.740, *p* < 0.001), supporting H4b, and on positive emotional arousal (DESPOS) (β = 0.521, *t* = 15.285, *p* < 0.001), supporting H4a. Furthermore, negative emotional arousal (DESNEG) significantly influenced behavioral intention to donate (BI) positively (β = 0.395, *t* = 7.759, *p* < 0.001), supporting H5b, as did positive emotional arousal (DESPOS) (β = 0.300, *t* = 5.791, *p* < 0.001), supporting H5a. The empirical evidence from these tests is consolidated in Table 6.

#### 4.3.1. Mediation Analysis

Mediation analysis was undertaken to ascertain the mediating roles of attitudes towards advertisements (AADs) and emotional arousals (DESPOS and DESNEG) in shaping the relationship between the personality traits of Honesty–Humility (HH), Emotionality (E), and Agreeableness (A) and behavioral intention to donate (BI). The results from the analysis highlighted the significant influences of these variables. The pathway from Honesty–Humility (HH) to behavioral intention to donate (BI) shows both direct (β = 0.087, *t* = 2.186, *p* < 0.05) and indirect effects (through AAD and DESPOS/DESNEG), with significant indirect effects supported (H6a: HH → AAD → DESPOS → BI, β = 0.031, *t* = 3.358, *p* < 0.001; H6a: HH → AAD → DESNEG → BI, β = 0.029, *t* = 3.274, *p* = 0.001). Given the presence of significant direct and indirect effects, this suggests partial mediation in the relationship between HH and BI through the mediated pathways of AAD and emotional arousal. For Emotionality (E) and Agreeableness (A), the direct effects on behavioral intention to donate (BI) were not significant (E → BI: β = 0.018, *t* = 0.435, *p* = 0.332; A → BI: β = 0.033, *t* = 0.811, *p* = 0.209), but both demonstrated significant indirect effects through AAD and emotional arousal (E → AAD → DESPOS → BI, β = 0.020, *t* = 2.370, *p* = 0.009; E → AAD → DESNEG → BI, β = 0.018, *t* = 2.347, *p* = 0.009; A → AAD → DESPOS → BI, β = 0.021, *t* = 2.414, *p* = 0.008; A → AAD → DESNEG → BI, β = 0.019, *t* = 2.567, *p* = 0.005). The absence of significant direct effects coupled with significant indirect effects indicates full mediation for Emotionality and Agreeableness in their relationship with BI through the mediators of AAD and emotional arousal. The detailed results are displayed in Table 7.

#### 4.3.2. Moderation Analysis

The study assessed the moderating role of message type on the relationship between Emotional Arousals (DESPOS and DESNEG) and behavioral intention to donate (BI). Without the inclusion of the moderating effect, the R^2^ value for BI was 0.341. This shows that the 34.1% change in BI is accounted for by DESPOS and DESNEG. With the inclusion of the interaction term, the R^2^ increased to 35.6%. This shows an increase of 1.5% in variance explained in the dependent variable (BI). The analysis sought to determine the influence of positive and negative emotional arousal (DESPOS and DESNEG, respectively) on BI, as well as to assess the moderating effect of message type, which was coded as egoistic (0) and altruistic (1). Positive emotional arousal had a significant positive impact on BI as DESPOS increases (β = 0.300, SE = 0.052, *t* = 5.791, *p* < 0.001). Similarly, negative emotional arousal was also found to positively affect BI with higher levels of DESNEG, indicating an increased intention to donate (β = 0.395, SE = 0.051, *t* = 7.759, *p* < 0.001). Concerning the moderating effects, the type of message significantly moderated the relationship between positive emotional arousal (DESPOS) and BI (β = 0.196, SE = 0.078, *t* = 2.531, *p* = 0.006). This implies that when the message is framed altruistically, the relationship between DESPOS and BI is stronger. Conversely, however, there was a significant negative interaction effect between message type and negative emotional arousal (DESNEG) on BI (β = −0.212, SE = 0.076, *t* = 2.773, *p* = 0.003), which indicated that egoistic messages increased the association between DESNEG and BI positively in support of H7. A moderation analysis summary is presented in Table 8.

**Figure 2:**
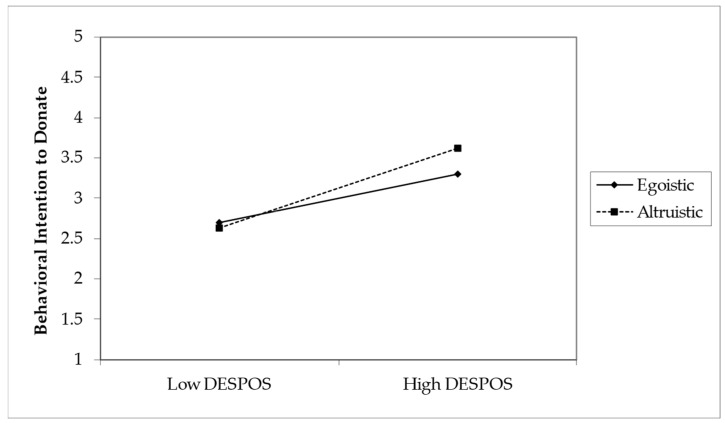
Two-way interaction effect of message type at different levels of DESPOS on BI.

**Figure 3:**
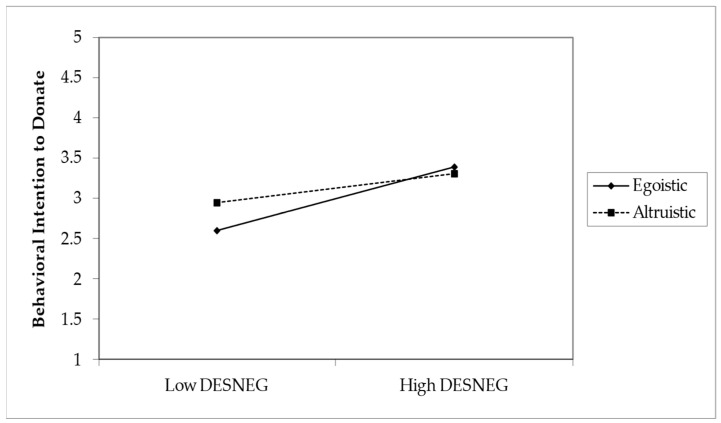
Two-way interaction effect of message type at different levels of DESNEG on BI.

Furthermore, a simple slope analysis is presented to help understand how the effects are moderated (Figure 2 and Figure 3). In the case of Figure 2, it implies that under low DESPOS conditions, the slope is much steeper for the altruistic message type compared to the egoistic one. This indicates that at low levels of DESPOS, the impact of an altruistic message on behavioral intention to donate (BI) is greater compared to an egoistic message. On the contrary, when DESPOS levels are high for egoistic message types, the slopes become less steep, indicating that enhanced positive emotional arousal does bring out intentions to donate as strongly when the message is egoistic. Likewise, in Figure 3, where there is an absence of negative emotional arousal (Low DESNEG), the slope with regard to altruistic messages has a more or less equal inclination compared to the egoistic message, indicating that the impact of an altruistic message on BI is lessened. However, at higher levels of negative emotional arousal (High DESNEG), BIs associated with altruistic messages begin to decrease downwardly. The above shows that increased levels of DESNEG significantly lower the efficiency of altruistic messages in relation to BI. In conclusion, higher positive emotional arousal enhances the impact of altruistic messaging on donation intentions, while higher negative emotional arousal diminishes this impact.

## 5. Discussion

In H1 through H3, we examined the direct relationship between HEXACO’s traits and BI, and a surprising finding was that Honesty–Humility was the only personality trait that demonstrated a statistically significant connection with behavioral intention to donate (BI), thus supporting H1. What we would anticipate from the literature are traits with high indices that highlight and are associated with individuals to perform endeavors of social change, compassion, and empathy, such as Agreeableness and Honesty–Humility [71,76,78], although there have been instances where Emotionality along with Agreeableness are not ideal or suitable indicators of prosocial predispositions due to the significant emotional fluctuations that these people exhibit [71,72]. This relationship can potentially be explained by the fact that individuals with high Emotionality demonstrate empathy and emotional comprehension; in our instance, the decision to donate in the future might have been based on rational rather than emotional motives. Another explanation is that these people are seeking for more logical or practical reasons to motivate them to engage in this type of behavior because they are less receptive to emotional pressures (or even guilt-laden messages), and thus, altruistic messages did not have the intended outcome. From our literature review, it is evident that altering the message framing can result in varied outcomes [36,46,47,81]. This becomes particularly apparent when the behavioral aspect is incorporated, such as personality factors that influence motivation, cognitive perception, and emotional stimulation, emphasizing the complexities of human behavior. Nonetheless, our findings highlight the significance of personality traits in the context of humanitarian donations, as well as the necessity of considering them when designing advertising campaigns that appeal to various audiences. Personality traits are complex and can interact with many aspects of cognitive and emotional processes. Due to their underlying motivations, cognitive procedures, and emotional dispositions, people react uniquely to various message types. These findings highlight the complexities of the relationship between personality traits alongside certain acts, such as blood donation. Furthermore, they serve as a reminder that a single personality characteristic is unlikely to fully predict behavioral patterns and decision-making processes. Elements such as individual values, beliefs, cultural standards, social norms, and external influences, all have a major influence on future blood donation intentions.

In H4a and H4b, when investigating the relationship between attitude towards the ad (AAD) and emotional arousal, the findings revealed a direct and positive relationship between AAD and DES in both positive and negative emotional arousals, a verdict that extends to previous findings as we observed coherence across emotional contexts [27,29,34]. This time, our participants appear unaffected by the type of triggered emotions, with AAD demonstrating a positive relationship with both DESPOS and DESNEG. It is evident that participants exhibit a favorable disposition towards advertisements that influence their perceived behavior and their response to the advertising content, thus highlighting the significance of emotional appeals and the overall influence of advertising in persuading people to be more socially engaged and proactive [42,61]. As a result, responsible social organizations that strive to attract new individuals to donate blood voluntarily must develop their advertising strategies to resonate with the public’s perceptions and positive attitudes for encouraging behaviors such as donating blood. Advertisement perception plays a central role in shaping attitudinal and behavioral intentions, as well as providing critical information for effective campaign design [42,44,90].

When examining the relationship of both subscales of Emotional Arousal (DESPOS and DESNEG) with BI, the results showed that there is a positive direct association between DESPOS and DESNEG and BI, thus supporting H5a and H5b. This positive connection demonstrates once again that people who experience significant positive emotions are far more inclined to participate in altruistic attitudes. In the context of the positive emotional process, it emphasizes the impact of emotional experiences on establishing intentions that encourage the desire to contribute to the benefit of society in general and others as well. The statistically significant association with negative emotions indicates that, in the context of our experiment, negative emotional arousal proved to have a crucial part in the decision-making process. This suggests that that regardless of the stated emotion (positive or negative) and their motivational power, it can be a predictive force in behavioral intentions considering self-oriented actions [47,81]. Strong evoked emotions function emerged as a motivator for prosocial behaviors such as volunteering, highlighting the importance of emotions in influencing behaviors [6,136]. The complexities provided by emotional reactions are not always apparent or expected, especially when personal experiences and individual perception have a tangible impact on emotional swings, as appears to be the case in our scenario, where both types of emotions seem to influence participant engagement. It is also important to note that this relationship is positive, which may indicate the effect of emotions on the change in altruistic dispositions, as people who experience strong emotional changes tend to be more sensitive and easily motivated to perform acts of solidarity such as volunteering. Further investigation is necessary to determine the complexities of this relationship. It would be interesting to investigate if this connection is affected by other moderating factors such as social norms, age, or other situational contexts that could potentially have a direct impact on their altruistic attitude in the future. There is a compelling case for egoistic motives in blood donation behavior as it can be shaped from internal volunteering goals, aspirations, and benefits. We have already discussed that selfish drives underline altruistic behaviors; thus, a balance of emotions can act as decision catalysts and evoke emotional responses that motivate individuals to consider self-oriented actions, such as donating blood. This finding expands our understanding of the interconnections of different emotional arousals and how they can influence the fine line between altruistic and egoistic choices.

Moreover, via H6a, H6b, and H6c, we investigated whether the relationship between the HEXACO personality traits and BI is mediated by attitudes towards an advertisement (AADs) and emotional arousal. The results from the mediation analysis revealed a full mediation for Emotionality and Agreeableness, suggesting that in our case, the emotional reactions to advertisements fully account for the influence of these traits on donation intentions. People who are prone to sudden emotional swings are more inclined to avoid circumstances that cause psychological discomfort. Emotionality is a personality trait that defines where a person falls on the emotional spectrum in terms of emotional stability and responsiveness to emotional stimuli. Individuals with prominent levels of Emotionality possess emotional resilience in the face of adversity and are more likely to overcome challenges calmer and with self-control. Given that the design of these advertisements is aimed to establish an intense emotional stimulation of negative emotions, using images that evoke disgust (with the use of a needle), fear (a car accident and the presence of blood), and guilt, an association with this particular personality trait is to be expected [69,71,78]. As we have previously mentioned, for a significant segment of the general population, voluntary blood donation can be an intense and emotionally demanding process that can be overwhelming or anxiety-inducing, especially for people who exhibit great emotional fluctuations or intensity, often regardless of the levels of empathy and sensitivity they exhibit, which justifies the positive correlation of this personality trait with AAD. This finding emphasizes the intricate relationship between this personality trait and multifaceted behaviors such as blood donation, where psychological responses can influence people’s desire to donate blood, thus offering a valuable target for intervention strategies. In the context of this study, the fact that the full mediation pathway of the two traits Emotionality and Agreeableness was not directly associated with BI reinforces the opinion that the power of an advertisement encompasses not only emotional impact but also a cognitive appraisal of the message that aligns with a person’s beliefs and values. All of the above provide a roadmap for developing more efficient and effective blood donation campaigns that speak to the hearts and minds of potential donors.

With H7, the current study attempts to investigate the moderating effect of message type in mediating the effectiveness of emotional arousal and behavioral intention to donate. The results from the slope analysis indicate that different forms of appeal, altruistic versus egoistic, have diverse effects on increasing or decreasing individuals’ intent to give blood, depending on the nature of emotional arousal. This research demonstrates that when combined with positive emotions, altruistic messages can significantly enhance one’s willingness to donate blood, unlike egoistic messages coupled with negative emotions. As part of our research investigation, we addressed the critical role message framing and emotions related to behavior intentions. Positive emotional arousal could potentially increase the acceptance for altruistic messages that encourage blood donations as a social value, as opposed negative emotional arousal, which can result in psychological resistance and reduce the motivation for participation through selfish appeals. Additionally, the findings support the empathy–altruism theory, suggesting that positive emotions can promote altruistic acts when messages reflect social benefits, increasing intrinsic reward for the individual [88]. Despite their distinct motivations (personal benefit or altruism), their perceptions about the value of the displayed advertisement plays a crucial role in their future decisions, which can significantly impact the effective advertising strategies when it comes to blood donation campaigns [42,44,61,90]. In the egoistic context, it is indicated that the message’s framing and an individual’s goals are in behavioral equilibrium. When the message’s egoistic appeals match an individual’s egoistic tendencies, it has the potential to favorably influence their opinions, driving them to be more receptive to engaging in the suggested behavior. Another explanation is that the empathy and compassion that these people have as their main characteristics prompt them to be more open-minded towards selfish motives, ignoring the potential conflict behind this type of messaging [36,42,49,61,62]. When organizations conduct strategic planning for the optimum and effective communication of messages, they enhance their impact on a broader variety of consumer audiences, providing incentives not solely to contemplate but also to embrace the act of blood donation. The necessity of proper framing in advertising messages cannot be overstated in the field of volunteer blood donation, where each small act of charitable giving can save a life [42,61,137]. The motivations and intents of individuals to contribute to the well-being of others as a rewarding, transcending self-interest experience are expressed in the formation of positive attitudes. When these messages are carefully planned to resonate with people’s beliefs and sensitivities, they promote active commitment and community sustainability.

To conclude, our research supports and extends existing theories related to the pivotal role of emotions in motivating behavior. The direct and indirect paths and, most importantly, both positive and negative emotional arousal can increase the participants intention to donate, gives credit and reinforces the dual-process models of persuasion. This is a theory that elaborates on the impact of how both affective and cognitive routes can potentially lead to attitudinal and behavioral changes [7,138]. Additionally, our results suggest that positive emotions enhance people’s thought-to-action process and repertoires, like the propensity to engage in altruistic acts like blood donation, which provides concrete evidence and aligns with the broaden-and-build theory [138]. Our research sheds light on the influence of personality dimensions and specifically on altruistic behavior. With the integration of the HEXACO model, our study enhances the understanding of different traits and their predictive power in prosocial behaviors. Our findings demonstrate the importance and representative aspect of the Honesty–Humility trait, as the sixth personality dimension and unique to HEXACO, which encourages further theoretical exploration to uncover its predictability in the scheme of persuasive health communications and altruistic dispositions, as research on this is limited [71,72,139]. Our emphasis on message framing and the results can be useful guidelines for tailoring messages and strategies to elicit the desired behavioral outcomes. The significance of altruistic message framing on behavioral intentions under positive emotional arousal aligns with the empathy–altruistic hypothesis [2,7,88]. Altruistic appeals can act as external cues to evoke empathic concern and consequently altruistic intentions. Moreover, under negative emotional arousal, the effectiveness of egoistic message framing was reduced, which suggests that people strive for consistency between their beliefs, attitudes and behaviors [47,49,51,81]. The combination of negative arousal and egoistic messaging can potentially lead to higher dissonance and affect donation intentions.

This research is not without limitations. We studied personality traits to gain insights into individuals’ psychological traits as part of our comprehensive examination of the variables that influence our future intention to donate blood. We specifically utilized the HEXACO model; however, it is crucial to highlight that we did not investigate the impact of certain facets within these traits in this study, which can provide a distinct and more sophisticated view on personality. Our major focus was on three key personality characteristics: Honesty–Humility, Agreeableness, and Emotionality. While one of these three dominating traits was statistically significant, it is critical to investigate why Agreeableness and Emotionality, two of our anticipated personality traits, failed to demonstrate significance, particularly in the context of volunteering behavior [33,82]. Moreover, we intend to expand our study to non-student groups in the future. This is especially significant because little to no effort has been made to research Greek people’s pro-social acts, attitudes, and predispositions towards blood donation. Furthermore, we recognize that a number of additional factors, such as past behavior, socio-political circumstances, and demographic characteristics (e.g., gender, educational background), can influence blood donation [33,82]. Finally, since in our methodology, past and present donor behavior were not considered as independent variables, and instead we focused on the effect emotional appeals and message framing, in the future, we intend to incorporate these variables as moderators to expand our research.

## 6. Conclusions

Voluntary blood donation should bridge theoretical and philosophical boundaries in the hope of actively addressing current demands and challenges. Altruism, whether pure or ideological, is a fundamental motive towards an individual’s selfless engagement without rationales in charitable giving. People’s desire to participate in solidarity and social welfare is prompted and nurtured by need, regardless of personal advantage. Identifying donor motives, predispositions, and barriers and the determinants of delay or inactivity in donating blood is crucial for developing effective campaigns. It is essential to tailor recruiting techniques depending on population demographics, national healthcare system structures, and available resources. These initiatives should strive to recruit and retain blood donors by addressing various criteria and balancing supply and demand. In this effort, young adults belonging to the 18–30 age group feature as the primary target group for blood donation campaigns.

This study examined how emotions triggered by advertisements and the role of incentives, through message framing, affect the intention to donate blood. Attitudes towards advertisements and emotional arousal mediated the link between personality traits, especially Honesty–Humility, and the intention to donate. The type of message (egoistic vs. altruistic) also influenced the relationship between emotional arousal and donation intention. In conclusion, it is important to implement targeted emotional appeals in blood donation campaigns for the effective recruitment of potential donors.

These findings can have important practical implications for the development of effective social marketing campaigns. Based on our research, emotional appeals have a considerable effect in social marketing initiatives. An awareness of how emotions influence behavioral intentions assists marketers to develop emotionally resonant messages that elicit desired responses. Campaigns that are tailored to evoke specific reactions, whether positive or negative, can effectively engage audiences and compel a call to action. In the case of non-profit and public organizations, utilizing emotional stimuli, such as positive emotional arousal associated with altruistic behavior, allows for the development of intriguing blood donation ads that inspire compassion and personal and societal accountability. These insights can significantly benefit transfusion centers aiming to refine their donor recruitment strategies. In addition, establishing the connection between emotional reactions and behavioral outcomes allows for an improvement in messaging strategies to address diverse social issues, increasing their efficacy and societal influence. As our findings demonstrate, personality traits serve an essential part in shaping responses to social marketing endeavors. Recognizing how certain traits, such as Honest–Humility, Emotionality and Agreeableness, influence attitudes and behavioral intentions provides marketers with valuable knowledge. Messages that are tailored to particular personality traits can increase audience receptivity and engagement. Understanding that people with high Honesty–Humility respond positively to altruistic-themed messaging, for instance, presents a potential channel for developing attractive advertisements that are relatable to their empathetic nature. Integrating personality traits into campaign designs enables more nuanced and targeted approaches, increasing the effectiveness of social marketing campaigns while cultivating significant connections with varying audience segments. In summation, these findings can significantly contribute to understanding how diverse combinations of appeal strategies can potentially influence prosocial decisions and lead to a broadened understanding of blood donation behavior.

## Figures and Tables

**Table 1:** Experiment design [104].

Emotional Arousal		Type of Message	
		Altruistic	Egoistic
Negative	Fear	Ad01a	Ad01e
	Guilt	Ad02a	Ad02e
	Disgust	Ad03a	Ad03e
Positive	Inspiration	Ad04a	Ad04e
	Interest	Ad05a	Ad05e
	Joy	Ad06a	Ad06e

**Table 2:** Sample profile.

		Frequency	Percentage
**Gender**	Male	222	48.1%
	Female	240	51.9%
**Age**	18–25	140	30.3%
	26–30	119	25.8%
	31–40	101	21.9%
	41–50	70	15.2%
	51–60	23	5.0%
	60+	9	1.9%
**Education**	High School Graduate	23	5.0%
	Undergraduate Student	165	35.7%
	Graduate	132	28.6%
	Postgraduate	77	16.7%
	Postgraduate Student	49	10.6%
	PhD Candidate	12	2.6%
	Doctoral	4	0.9%

**Table 3:** Factor loading reliability and convergent validity.

Construct	Items	Factor Loadings	Cronbach’s Alpha	rho_A	CR	AVE
Agreeableness	A1	0.560	0.570	0.577	0.753	0.436
	A2	0.681				
	A3	0.605				
	A4	0.776				
AAD	AAD1	0.787	0.797	0.797	0.868	0.622
	AAD2	0.814				
	AAD3	0.782				
	AAD4	0.772				
BI	BI1	0.773	0.792	0.801	0.879	0.708
	BI2	0.903				
	BI3	0.843				
DESNEG	DESNEG1	0.905	0.891	0.894	0.932	0.821
	DESNEG2	0.914				
	DESNEG3	0.900				
DESPOS	DESPOS1	0.787	0.606	0.644	0.784	0.552
	DESPOS2	0.817				
	DESPOS3	0.606				
Emotionality	E1	0.902	0.792	0.785	0.837	0.573
	E2	0.482				
	E3	0.792				
	E4	0.787				
Honesty–Humility	HH1	0.803	0.608	0.616	0.772	0.462
	HH2	0.577				
	HH3	0.646				
	HH4	0.674				

**Table 4:** HTMT ratio.

	A	AAD	BI	DESNEG	DESPOS	E	HH
A							
AAD	0.252						
BI	0.196	0.760					
DESNEG	0.264	0.430	0.542				
DESPOS	0.216	0.699	0.649	0.416			
E	0.439	0.177	0.160	0.155	0.211		
HH	0.138	0.277	0.180	0.089	0.137	0.118	

**Table 5:** Fornell and Larcker criterion.

	A	AAD	BI	DESNEG	DESPOS	E	HH
A	**0.661**						
AAD	0.166	**0.789**					
BI	0.133	0.608	**0.841**				
DESNEG	0.175	0.363	0.460	**0.906**			
DESPOS	0.124	0.521	0.467	0.292	**0.743**		
E	0.315	0.168	0.142	0.161	0.174	**0.757**	
HH	−0.027	0.198	0.129	0.052	0.064	0.006	**0.680**

The bold values represent the square root of AVE.

**Table 6:** Hypothesis testing.

Hypotheses	Path	Coefficient (β)	SD	*t*-Value	*p*-Value	Results
H1	HH → BI	0.087	0.040	2.186	0.014	Supported
H2	E → BI	0.018	0.042	0.435	0.332	Rejected
H3	A → BI	0.033	0.041	0.811	0.209	Rejected
H4a	AAD → DESPOS	0.521	0.034	15.285	0.000	Supported
H4b	AAD → DESNEG	0.363	0.042	8.740	0.000	Supported
H5a	DESPOS → BI	0.300	0.052	5.791	0.000	Supported
H5b	DESNEG → BI	0.395	0.051	7.759	0.000	Supported

**Table 7:** Mediation analysis.

Hypotheses	Direct Effects	Coefficient (β)	*t*-Value	*p*-Value	Results
	HH → BI	0.087	2.186	0.014	
	E → BI	0.018	0.435	0.332	
	A → BI	0.033	0.811	0.209	
	**Total Effects**	**Coefficient (β)**	***t*-Value**	***p*-Value**	
	HH → BI	0.148	3.238	0.001	
	A → BI	0.073	1.598	0.055	
	E → BI	0.056	1.196	0.116	
	**Specific Indirect Effects**	**Coefficient (β)**	***t*-Value**	***p*-Value**	
H6a	HH → AAD → DESPOS → BI	0.031	3.358	0.000	Supported
H6a	HH → AAD → DESNEG → BI	0.029	3.274	0.001	Supported
H6b	E → AAD → DESPOS → BI	0.020	2.370	0.009	Supported
H6b	E → AAD → DESNEG → BI	0.018	2.347	0.009	Supported
H6c	A → AAD → DESPOS → BI	0.021	2.414	0.008	Supported
H6c	A → AAD → DESNEG → BI	0.019	2.567	0.005	Supported

**Table 8:** Moderation analysis.

Relationship	Beta	SE	*t*-Value	*p*-Value
DESPOS → BI	0.300	0.052	5.791	0.000
DESNEG → BI	0.395	0.051	7.759	0.000
Moderating Effect (message_type × DESPOS) → BI	0.196	0.078	2.531	0.006
Moderating Effect (message_type × DESNEG) → BI	−0.212	0.076	2.773	0.003

## Data Availability

The raw data collected by the survey are available upon request to the corresponding author.

## Appendix A

**Table A1:** Measurement items used for data analysis.

Attitude towards the Ad (Five-Point Semantic Differential Scale) (AAD)		
AAD1	Like the ad (1 = I dislike the ad—5 = I like the ad)	Originally Holbrook and Batra [114] Adapted from Ranganathan and Henley [115]
AAD2	Favorable (1 = I react unfavorably to the ad—5 = I react favorably to the ad)	
AAD3	Positive (1 = I feel negatively toward the ad—5 = I feel positive toward the ad)	
AAD4	Advertisement is good (1= the ad is bad—5 = the ad is good)	
AAD5	Information (1 = The ad is uninformative—5 = informative)	
**Emotional Arousal (DES)**		
**Positive Emotions (DESPositive)**		
DES_GLAD	I felt glad, happy, joyful.	Galanakis, Stalikas, Pezirkianidis and Karakasidou [113]
DES_INTER	I felt interested, alert, curious.	
DES_INSP	I felt inspired, uplifted, elevated.	
**Negative Emotions (DESNegative)**		
DES_SCARE	I felt scared, fearful, afraid.	Galanakis, Stalikas, Pezirkianidis and Karakasidou [113]
DES_DISG	I felt disgust, distaste, revulsion.	
DES_SCORN	I felt contemptuous, scornful, disdainful.	
**Behavioral Intention (BI) to Volunteer (5-point scale) (BI)**		
BI1	Based on the advertisement I just saw, I would unlikely/likely volunteer and donate blood to the organization sponsoring the ad.	Lim, Bouchacourt and Brown-Devlin [116]
BI2	Based on the advertisement I just saw, I would possibly/probably volunteer and donate blood to the organization sponsoring the ad.	
BI3	Based on the advertisement I just saw, I would uncertainly/certainly volunteer and donate blood to the organization sponsoring the ad.	
